# Ephemeral Keys Authenticated with Merkle Trees and Their Use in IoT Applications

**DOI:** 10.3390/s21062036

**Published:** 2021-03-13

**Authors:** Pavol Zajac

**Affiliations:** Faculty of Electrical Engineering and Information Technology, Slovak University of Technology in Bratislava Ilkovičova 3, 812 19 Bratislava, Slovakia; pavol.zajac@stuba.sk

**Keywords:** ephemeral keys, Merkle tree, authenticated KEM

## Abstract

Public key algorithms based on quasi-cyclic binary moderate-density parity-check codes (QC-MDPCs) and QC low-density parity-check codes (QC-LDPCs) codes for key encapsulation and encryption submitted to the NIST post-quantum competition (Bit Flipping Key Encapsulation (BIKE), QC-MDPC KEM, LEDA) are vulnerable against reaction attacks based on decoding failures. To protect algorithms, authors propose to limit the key usage, in the extreme (BIKE) to only use ephemeral public keys. In some authenticated protocols, we need to combine each key with a signature, which can lead to increased traffic overhead, especially given the large signature sizes of some of the proposed post-quantum signature schemes. We propose to combine ephemeral public keys with a simple Merkle tree to obtain a server authenticated key encapsulation/transport suitable for TLS-like handshake protocols. This allows a very simple public key verification on the client, leading to efficient protocols suitable for Internet of Things applications.

## 1. Introduction

The fast progress of quantum computing brings new significant threats to Internet of Things applications. Standard symmetric cryptographic algorithms need to increase key sizes to resist attacks based on the applications of Grover’s algorithm [1]. While the current encryption standard AES should still be secure [2], this might not hold in general for many lightweight IoT ciphers, such as [3], with a low number of AND gates, due to (quantum) algebraic attacks [4].

An even worse situation happens when considering public key cryptography, as most of the current cryptographic primitives such as RSA, DSA, ECDSA, the Diffie–Hellman protocol, and others are considered broken when the attacker has access to a quantum computer. This is due to the polynomial-time factoring algorithm of Peter Shor [5] and subsequent algorithms that attack the discrete logarithm problem in finite fields and elliptic curve groups. Fortunately, there is a large number of public key algorithms that can resist quantum attacks, such as the McEliece algorithm [6]. The area of cryptography that studies quantum-resistant algorithms is called post-quantum cryptography.

Note that there is still a large number of practical considerations when trying to adopt post-quantum algorithms for practical areas of interest, especially for IoT applications. Algorithms, such as McEliece, have often large keys and unbalanced performance for encryption/decryption or signing/verification [7]. There is also a concern for the secure implementation of such algorithms that do not leak side-channel information. We study such post-quantum applications [8] in the area of secure group establishment [9]. In the current paper, we focus on the question of post-quantum public key verification.

The recent NIST call for public key post-quantum cryptographic algorithms [10] has motivated many researchers to propose new cryptographic schemes that are believed to be secure against quantum adversaries. One of the proposed candidates for key encapsulation is a suite of algorithms named Bit Flipping Key Encapsulation (BIKE) by Aragon et al. [11]. In the third round of the competition, it was selected in the suite of “Alternate Candidates: Public key Encryption and Key-establishment Algorithms” [12].

BIKE algorithms are based on quasi-cyclic binary moderate-density parity-check codes (QC-MDPCs). A basic QC-MDPC KEM was also proposed by Yamada et al. [13]. Another proposed system, LEDA by Baldi et al., is based on QC low-density parity-check codes (QC-LDPCs). The class of algorithms based on QC-LDPC and QC-MDPC can achieve post-quantum security with parameters similar to the currently used RSA cryptosystem.

One of the main problems with LDPC/MDPC based designs is the probabilistic nature of decoding algorithms. The non-negligible decoding failure rate can lead to various attacks that can derive the secret key, such as [14,15,16]. To prevent these attacks, BIKE relies on generating one time ephemeral keys. The authors of QC-MDPC KEM also recommend using only ephemeral keys. LEDA limits the number of uses for each key pair, but the number of key uses might be lower than originally proposed due to new attacks, such as Fabsic et al. [17].

In the TLS 1.3 handshake protocol [18], an initial session key is negotiated in key exchange using ephemeral public keys. The handshake is authenticated by the server signing protocol messages and verified by the client using the server’s certificate. Instead of (EC)DHE, a post-quantum public key cryptosystem or KEM with ephemeral keys, such as BIKE, QC-MDPC KEM, or LEDA, can be used to transport initial key shares to provide post-quantum security. To fully migrate TLS handshakes to the post-quantum era, we also need to replace server-side signatures and certificates with quantum-resistant routines. The signature structure can be compatible with the previous TLS 1.2 handshake with signed keys.

There are multiple candidates for post-quantum signatures with varying public key and signature sizes. In a typical handshake scenario, both a public key and a signature need to be sent. Among balanced-size algorithms, the Falcon signature [19] scheme (lattice-based) provides Level-1 security with a 897 byte public key and a 617 byte (on average) signature size. Short signatures are achieved by multivariate proposals, led by HiMQ-3F [20], with 67 bytes needed for signature and 100,878 bytes for the public key. On the opposite side, hash-based signature schemes provide short public keys and long signatures, led by Sphincs+ [21] with a 32 byte public key and an 8080 byte signature.

Hash-based signature schemes are based on one time signatures (OTSs) that are combined with Merkle trees [22] or Goldreich trees [23] to provide multiple-time signatures. Our observation is that when we use ephemeral public keys, we already have a single-use key pair with its corresponding secret key. Thus, we do not need to produce a specific one time signature; we only need to pre-authenticate the sequence of public keys. We propose to use Merkle trees with leaves directly based on hashes of (precomputed) ephemeral public keys. An *l* level Merkle tree will allow us to authenticate $2^{l}$ ephemeral trees, with the public key size equal to the size of a single hash value and another l−1 hash value needed for the signature (to store the path through the Merkle tree). For example, an l=20 level Merkle tree will provide authentication for $2^{20}$ ephemeral keys, with the total signature + public key (PK) size for a 128 bit security level equal to 640 bytes. This comes at the additional cost of storing the Merkle tree on the server with $2^{l}$ hashes (32 MB in the previous setup).

If the number of one time certificates is not sufficient, with PKI support, the root of the Merkle tree can be signed by the CA with an algorithm with a different trade-off. As the public key of the CA can be pre-installed in the client device, the CA’s algorithm can have a short signature size and a large public key. Different trade-offs with the sizes of Merkle trees and certification paths can be made for different usage scenarios (depending on the number of accesses, available storage, tree precomputation time, etc.), e.g., we can construct another simple Merkle tree on top of $2^{20}$ OTSs on the server as a Level 1 local certification authority (whose root is further signed by a real authority). This will provide a flexible server setup with $2^{40}$ usable keys.

On the other hand, in IoT applications, we do not need an extra PKI layer to authenticate the keys, as the Merkle tree root can be preinstalled in the client devices at a negligible cost. As the root of the tree is a single hash, it suffices to store 32 bytes if the 128 bit security level is sufficient.

The stateful authentication of public keys based on a Merkle tree retains the forward secrecy because we can derive private keys on the fly from a secret seed value using a one way function (old private keys are not needed anymore after they are used). We can modify the scheme to become stateless if we relax conditions on the key reuse (e.g., for use with the LEDA cryptosystem [24]). In this case, however, we will lose the forward secrecy, because we must store the private keys, or the pre-key, that can be used to derive any private key authenticated in the Merkle tree.

## 2. Preliminaries

We work in the client-server scenario typical for Internet communication. We rely on standard cryptographic tools and primitives:

- Public key cryptosystem with a pair of keys: We are only interested in key generation and authentication. The KeyGen primitive for a cryptosystem should efficiently generate a pair (SK,PK) (from some randomness; see further), where SK denotes a secret key, and PK a public key. We suppose that to initiate secure communication between the client and server, it is sufficient to provide a mechanism to transport the authenticated public key of the server to the client. We are not interested in further protocols that realize the rest of the secure channel establishment, etc.
- The KeyGen primitive can be based on a deterministic algorithm $KDF:Z_{2}^{n}\to K$, that computes keypair (SK,PK) from a bitstring *k* of length *n*. We call *k* a pre-key. In the classical setting, n=λ, where λ is a security level, but in the post-quantum setting, we use pre-keys n=2λ to prevent Grover’s algorithm-based speedup.
- A truly random pre-key is required for a secure public key system. In our scheme, we use a single master (secret) pre-key that is generated as a true random bit-string. All other pre-keys are derived with a one way function $OWF:Z_{2}^{n}\to Z_{2}^{n}$.
- In the construction of public key authentication, we also use a specific cryptographically secure hash function denoted by hash (in practice, instantiated by the standard SHA-2 or SHA-3). Both OWF and KDF can be implemented with the correct use of the same hash function (or by a different specific mechanism, as required by the system/protocol).

## 3. Merkle Tree

The Merkle tree was introduced by Merkle [22] to allow signing arbitrary documents with any one way function *F*. It is the basis of post-quantum secure hash-based signatures. The Merkle tree is used in conjunction with one time signature (e.g., Lamport [25] or Winternitz [26]) and extends them for an arbitrary number of uses.

We do not require a full hash-based signature scheme in our use case. Instead, we use a simple Merkle tree method. A Merkle tree is based on a binary tree with *l* levels. Let $h_{1}$ denote the root of the tree. Let each node $h_{i}$ have a left sub-node $h_{2i}$ and a right sub-node $h_{2i+1}$. The Merkle tree is a defined by marking each node of the tree by a hash of subnodes as follows: $h_{i}=hash\left(h_{2i}|h_{2i+1}\right)$.

Our goal is to use the Merkle tree for authentication. For this purpose, it is sufficient to make the root node $h_{1}$ publicly known. Now, we can prove for any $h_{j}$ that its associated value is indeed a part of the precomputed Merkle tree without publishing the whole Merkle tree. Indeed, for every *j*, there exists a path from $h_{1}$ to $h_{j}$ in the Merkle tree. Suppose that this path is $h_{1},h_{i_{1}},h_{i_{2}},\ldots ,h_{j}$. To authenticate the hash value associated with $h_{j}$, we need to publish hash values only for those nodes that were used to construct the hash values of parent nodes on this path and that are not included in this path. Thus, the verification string of node $h_{j}$ consists of hashes associated with each missing sub-node $h_{2i}$ or $h_{2i+1}$ required to compute the $h_{i}$ associated with nodes on the verification path up to the Merkle tree root $h_{1}$.

**Figure 1:**
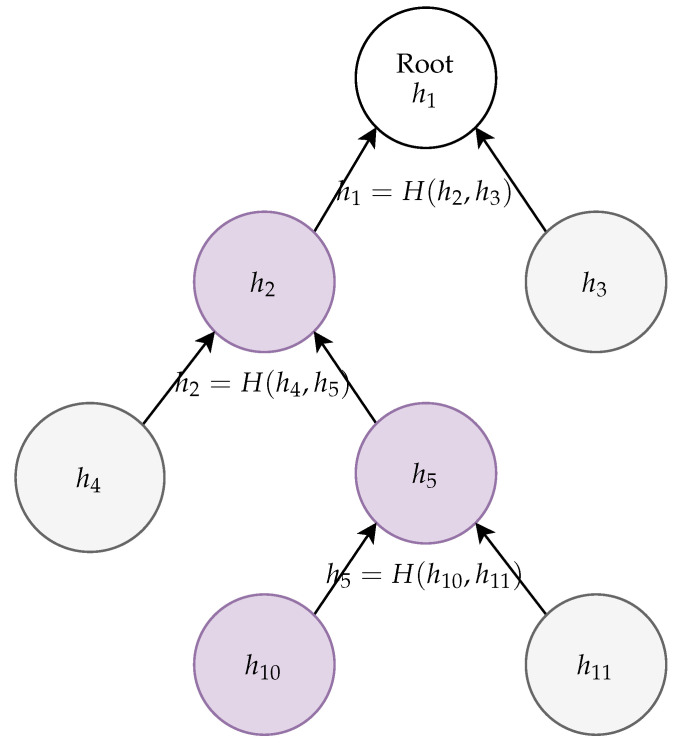
Example of a relevant part of a Merkle tree. The validity of $h_{10}$ can be verified, if we provide hashes $h_{11}$, $h_{4}$, and $h_{3}$: Firstly, we compute $h_{5}^{\prime }=hash\left(h_{10}|h_{11}\right)$, and then, $h_{2}^{\prime }=hash\left(h_{4}|h_{5}^{\prime }\right)$. Finally, we verify that $h_{1}=hash\left(h_{2}^{\prime }|h_{3}\right)$.

Suppose that $h_{i_{1}}=h_{2}$. We can verify that $h_{2}$ is indeed in the Merkle tree by providing a hash value associated with $h_{3}$ and checking that $h_{1}=hash\left(h_{2}|h_{3}\right)$. As $h_{1}$ is publicly known, we verify $h_{2}$ and can continue with verification recursively, using $h_{2}$ as the root node for recursive verification of its subnodes. An example of a Merkle tree verification is depicted in Figure 1.

Note that the Merkle tree is essentially infinite. In practice, we use a fixed number of levels, say *l*. Then, the final leaves have indexes between $2^{l}$ and $2^{l+1}-1$ (here, l=0, meaning a degenerate tree with only the root node). The verification starts from the bottom of the tree (on level *l*). For verification purposes, we need to calculate hashes on the path from the provided verification string of nodes directly connected to the path. Each verification string of individual nodes on level *l* consists of just *l* hashes, instead of the whole Merkle tree containing $2^{l}$ hashes.

## 4. Authenticating Ephemeral Keys with the Merkle Tree

In the TLS protocol, the server during handshake authenticates itself with a server certificate. This contains a signed server public key. Depending on the chosen suite of algorithms, the server public key is either used to encrypt the client secret, or it is only used to authenticate the ephemeral key used for Diffie–Hellman key exchange.

Current standard algorithms such as RSA or ECDHE are unfortunately not quantum-resistant and should be replaced by suitable post-quantum cryptographic schemes. The NIST post-quantum competition selected so far some suitable algorithms and their alternatives, which can replace current algorithms in TLS and other cryptographic protocols. The TLS 1.3 goal of forward secrecy can be achieved by using standardized post-quantum KEM with ephemeral keys. Such keys however still need to be authenticated by the server’s certificate, which also needs to be quantum-resistant.

Post-quantum signature schemes have significant disadvantages over currently used RSA or (EC)DSA signatures, namely the large public key size or signature size.

Our main idea is to replace full digital signatures used in ephemeral server key authentication with the use of the Merkle tree. We can understand this as a problem of authenticating a set of public keys, each of which can be used only once. The corresponding secret keys are never revealed, but their ownership is verified in the course of the underlying protocol.

The set of private keys can be randomly generated and stored in a centralized key store on a server. A more efficient option is to derive private keys from some master secret (a secret pre-key). In the pre-key approach, we can derive consecutive secret keys from the pre-keys chained in a sequence with a one way function OWF that derives the next pre-key from the previous. Alternatively, we can derive each secret key from a master pre-key and an index in the set of keys. The sequential generation has the advantage of forward secrecy: the server can safely remove pre-keys for the ephemeral keys that were already used. However, the price of forward secrecy is the issue with concurrency and state-keeping. The index method allows a (pseudo-)random selection of public keys (with no state and parallel access to the server).

### 4.1. Sequential Tree Authenticated Ephemeral Keys

To generate an authenticated set of ephemeral keys, the server does the following precomputation:

1. Generate a random secret seed $k_{0}\in Z_{2}^{\lambda }$.
2. Use a one way function OWF to define a sequence of derived pre-keys $k_{i}=OWF\left(k_{i-1}\right)$.
3. Generate $2^{l}$ ephemeral key pairs $\left(SK_{i},PK_{i}\right)$ from pre-keys $k_{i}$ using the defined KDF function.
4. Compute the hashes $h_{2^{l}+i}=hash\left(PK_{i}\right)$.
5. Compute the rest of the Merkle tree with $h_{j}=hash\left(h_{2j}|h_{2j+1}\right)$.
6. Publish (signed by the CA or delivered to devices by a trusted channel) the root $h_{1}$.
7. Store as an initial (secret) state: $S=(0,k_{0})$ and the hash path from $h_{2^{l}}$ to the root.

**Figure 2:**
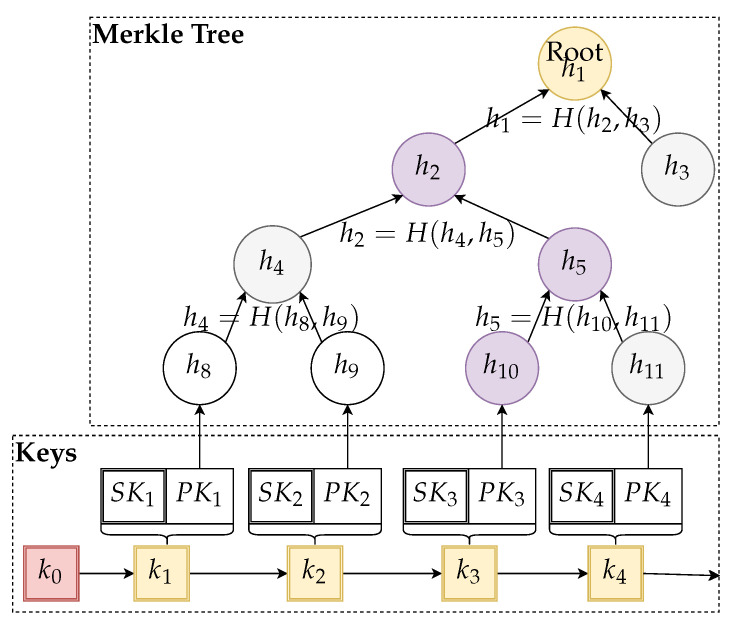
Example of a relevant part of a sequential key structure and Merkle tree authenticator built on top of the precomputed public key hashes.

The main idea of the algorithm with precomputed storage structures is depicted in Figure 2.

To initiate the KEM, the server does the following:

- Sends current public key $PK_{i}$ along with the verification string for the path in the tree from $h_{2^{l}+i}$ to the root.

To verify the authenticity of the public key, the client does the following:

- Verifies that $h_{2^{l}+i}=hash\left(PK_{i}\right)$ and that for each hash in the path to the root: $h_{j}=hash\left(h_{2j}|h_{2j+1}\right)$.

After the handshake part is finished and keypair $\left(SK_{i},PK_{i}\right)$ is no longer needed, the server prepares a new key pair from state $S=(i,k_{i})$:

1. Derive the next $k_{i+1}=OWF\left(k_{i}\right)$.
2. Recompute the hash path.
3. Store a new (secret) state: $S=(i+1,k_{i+1})$ and the hash path from $h_{2^{l}+i+1}$ to the root.

To recompute the hash path, there are two possibilities. The first option is that the whole Merkle tree is stored on the server. Note that the storage requirements grow exponentially with the height of the tree *l*. When we store the whole tree, generating a new signature is trivial. We just need to traverse the tree and look up the hashes that complement the path.

We can save some storage space by recomputing the required parts of the Merkle tree after the key use. We only need to store the current $k_{i}$ and the path from $h_{2^{l}+i}$ to the root. All the left-hand sub-nodes for the path from $h_{2^{l}+i+1}$ were already computed when verifying the path from $h_{2^{l}+i}$. Only the right-hand child nodes need to be recomputed, similar to the original Merkle tree precomputation. In this case, we never need to store $k_{j}$ with j<i; thus, old seed values can still be removed (to preserve forward secrecy).

Thus, to save storage, we do not need to store the (remaining) Merkle tree and the associated keys. On the performance side, this means that after each ephemeral key is used, the server needs to recompute all the remaining keys and the verification path for the next ephemeral key. However, if the key generation is too slow, we might prefer a compromise: store the remaining key-pairs, but recompute hash paths for each new verification path. This option, however, is more vulnerable to data-retention attacks and does not save much space (less than half of the space, depending on the ratio between ephemeral key size and hash size). For some algorithms, such as BIKE-2 [11], tree generation can be combined with more efficient batch key generation.

### 4.2. Parallel Tree Authenticated Ephemeral Keys

In some sensor and IoT applications, as well as other scenarios, values encrypted in the past are not of much use. In such a case, forward secrecy is not needed. Furthermore, we might also relax the key reuse requirements and only demand that one “ephemeral” key is not used too often (key reuse limitation).

In such cases, we can use a more efficient version of the tree authentication as follows.

To generate an authenticated set of ephemeral keys, the server does the following precomputation:

1. Generate a random secret seed $k_{0}\in Z_{2}^{\lambda }$.
2. Define pre-keys with $k_{i}=OWF\left(k_{0}|i\right)$, for $i=1,2,\ldots ,2^{l}$.
3. Generate $2^{l}$ ephemeral key pairs $\left(SK_{i},PK_{i}\right)$ from pre-keys $k_{i}$ using the defined KDF function.
4. Compute the hashes $h_{2^{l}+i}=hash\left(PK_{i}\right)$.
5. Compute the rest of the Merkle tree with $h_{j}=hash\left(h_{2j}|h_{2j+1}\right)$.
6. Publish (signed by CA or delivered to devices by a trusted channel) the root $h_{1}$.

**Figure 3:**
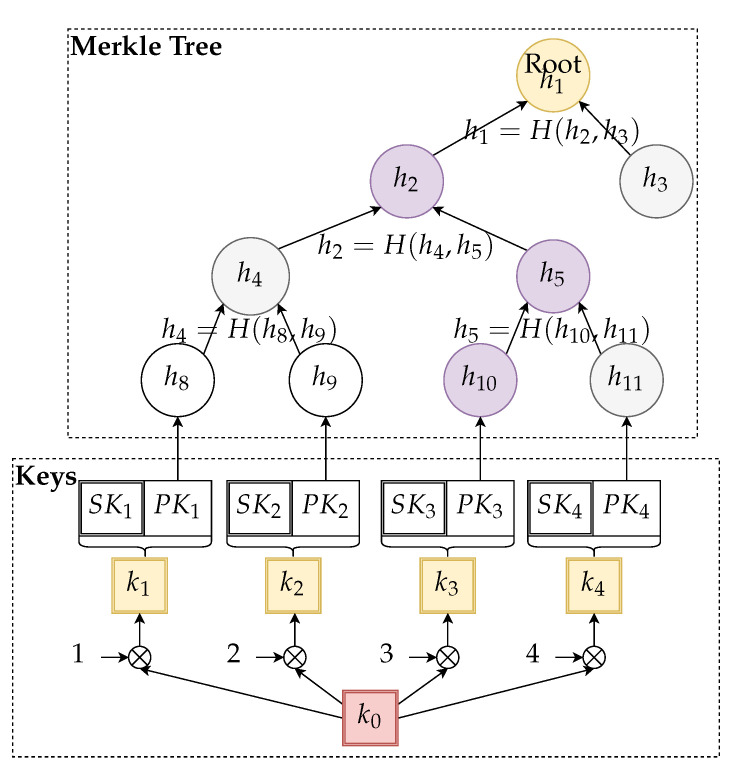
Example of a relevant part of a parallel key structure and Merkle tree authenticator built on top of the precomputed public key hashes.

The main idea of the algorithm with precomputed storage structures is depicted in Figure 3.

To initiate the KEM, the server does the following:

1. Selects random *i* from the set $\left\{1,2,\ldots ,2^{l}\right\}$.
2. Computes pre-key $k_{i}=OWF\left(k_{0}|i\right)$.
3. Generates ephemeral key pairs $\left(SK_{i},PK_{i}\right)$ from pre-key $k_{i}$ using the defined KDF function.
4. Sends public key $PK_{i}$ along with the verification string for the path in the tree from $h_{2^{l}+i}$ to the root.

To verify the authenticity of the public key, the client does the following:

1. Verifies that $h_{2^{l}+i}=hash\left(PK_{i}\right)$ and that for each hash in the path to the root: $h_{j}=hash\left(h_{2j}|h_{2j+1}\right)$.

In this scenario, we need to store the whole Merkle tree (a preferred version) or recompute it on demand. The random key selection limits a potential number of uses of a certain public key by a factor of $2^{l}$.

The main advantage of the parallel version is the state-less nature of the protocol, suitable for client-server scenarios with multiple parallel client connections. Although this scenario is also manageable in the sequential version, it needs more overhead (e.g., using a specific cache for keys and paths that are still in use). Furthermore, the sequential scenario assumes that there is only a limited number of pre-authenticated ephemeral keys. After all of the keys are used, we need to build a new authentication tree and redistribute the root of the tree. In the parallel version, we only need to rebuild the tree and redistribute the key, if the number of key reuses reaches the prescribed security limit.

Unlike the sequential version, the parallel version of the protocol does not inherently provide forward secrecy. If the attacker compromises the server and extracts $k_{0}$, she/he can reconstruct all past keypairs by using $k_{i}=OWF\left(k_{0}|i\right)$, followed by the computation of $\left(SK_{i},PK_{i}\right)$ from $KDF(k_{i})$.

We can introduce forward secrecy to the parallel protocol by a modification of the data storage. In this case, we need to store all precomputed keys in a large secure storage, instead of storing $k_{0}$. The pre-key $k_{0}$ is safely removed after all keypairs are precomputed. Thus, in the KEM initialization phase, the pre-key is not used at all; instead, a valid keypair $\left(SK_{i},PK_{i}\right)$ to be used in the next session is taken directly out of the secure storage. Once the pre-generated key pair $\left(SK_{i},PK_{i}\right)$ is used in a session, the secret key $SK_{i}$ needs to be securely deleted, and the position should be marked as invalid. If random *i* (selected in the initiation phase) points to an invalid key pair, *i* is incremented until a valid position is found. If the key storage is compromised, past keys are already removed, and the attacker cannot break already finished sessions.

## 5. Security Analysis

In this section, we analyze the security of the proposed scheme. While this is not a full formal analysis, we believe it is sufficient in the context of the general proposal due to the properties of the underlying building blocks. However, for specific uses in the more complex protocols, authors should analyze their design with respect to the whole scheme.

We base our analysis on the standard security notions based on challenger-attacker security games. An attacker is any probabilistic polynomial time (PPT) algorithm, which has a goal to win some proposed challenge game. In our scenario, the attacker can present any public key PK with an arbitrary authentication path $\left(H_{1},H_{2},\ldots ,H_{M-1}\right)$. The attacker “wins” if the PK gets accepted by the verifier. Our scheme provides a public key authentication, if any PPT attacker has only a negligible chance to win the challenge game. As usual, negligible chance means that the probability of winning the game is less then 1/poly(λ) for all λ greater than some $\lambda_{0}$, for any polynomial function poly.

Let us analyze the hypothetical situation when the attacker wins the challenge. This happens only when after evaluating the hash path with the initial h(PK), the verifier gets value HM. It is easy to see that if the attacker provided a valid HM to the verifier, he/she is able to create a preimage of a hash function *h* in some of the points of the original Merkle tree. The security of the scheme is reduced to the security of the used hash function.

Let us do a more in-depth analysis. For the sake of simplicity, let us suppose that the authentication path is always evaluated from the right side. The attacker’s PK and the sequence $\left(H_{1},H_{2},\ldots ,H_{M-1}\right)$ induce a sequence of hash values $g_{0}=h\left(PK\right)$, $g_{i}=h\left(g_{i-1}|H_{i}\right)$. The attacker’s success means that $g_{M}=h_{1}$. This means that either the attacker has constructed a preimage for $h_{1}$ or that $g_{M-1}=h_{2}$ and $H_{M-1}=h_{3}$, respectively. We can now repeat the argument to finding the preimage of $h_{2}$,$h_{4}$, etc., up to $h_{2M}$, which is the hash of an original public key in our database, which was authenticated by the Merkle tree. We have thus established that if the attacker succeeds, he/she has constructed a preimage for the function *h*. Thus, we can conclude that the authentication property of the scheme reduces to preimage resistance of the used hash function.

In the basic analysis, we ignored that the Merkle tree contains exponentially many nodes related to the parameter *M*. Thus, the situation, in this case, is slightly more complicated: an attacker can try to create a preimage to any of the $2^{M+1}$ hash values included in the Merkle tree. In the ideal case, the probability of creating a preimage with random tries is $2^{M+1}/2^{n}$, where *n* is the length of the hash. Thus, the attacker needs to test approximately $2^{n-M}$ values to succeed with at least 50% probability. Note that the attacker does not need to generate as many public keys, as he/she can try to combine hashes of randomly generated public keys with hashes already in the public Merkle tree. However, this optimization does not reduce the expected work factor, expressed as the number of hash calls. If hash size n=2λ, where lambda is the chosen security level, the Merkle tree can contain up to M=λ levels to resist preimage attacks (the work factor is $2^{n-M}=2^{2\lambda -\lambda }=2^{\lambda }$). In practice, *M* is significantly lower than λ (due to practical size constraints on the database of public keys), and the generic attack is not possible. We give a formal argument in the next section (Section 5.1).

### 5.1. Formal Security

In this section, we formalize the security of the proposed scheme. Firstly, let us summarize the security assumptions. We base our scheme on the IND-CPA (indistinguishable under chosen plaintext attack) secure public key encryption system defined by algorithms (Gen,Enc,Dec). IND-CPA security means that any PPT adversary has only a negligible advantage when trying to distinguish between encryption of two chosen plaintexts. One corollary of IND-CPA security is that it is infeasible to compute secret key SK from a corresponding public key PK (otherwise, the attacker would just compute SK and decrypt the challenge ciphertext).

Furthermore, we use a secure hash function hash to build the Merkle tree. We require that hash be (at least) preimage resistant. This means that any PPT adversary has only a negligible probability of producing any value *x* that hashes to a known hash value *y*. When our security level is λ, we require that $Pr\left(A\left(1^{\lambda }\right)=x:hash\left(x\right)=y\right)\leq 2^{-\lambda }$ for any PPT attacker A.

In our schemes, ephemeral key pairs are derived from pre-keys by using the key derivation function KDF. We model KDF by a pseudo-random generator (PRNG) with *n* bit seed, where *n* is determined depending on the parameters of the scheme. We require that PRNG output be indistinguishable from a random bit string. This means any PPT attacker’s advantage in guessing whether the output is random or produced by our PRNG bounded by $2^{-n}$.

Our security goal is to provide key authentication for the underlying public key scheme. This means that we must not compromise the IND-CPA property and add a property that only a server that established trust with the challenger before the game starts can decrypt messages. We can model these security goals by the following security game.

The challenger is instantiated with a Merkle tree root $h_{0}$. We suppose that the whole Merkle tree, as well as pre-authenticated public keys $PK_{i}$’s are available for the attacker. The attacker does not have access to $SK_{i}$’s or to pre-keys.

During the security game, attacker A sends two chosen messages $m_{0},m_{1}$ to the challenger, along with a public key PK and its authentication path ${\left\{h_{i_{j}}\right\}}_{j=1}^{M}$. The challenger verifies the authenticity of the received PK by computing hashes following the authentication path. He/she generates a random bit *b*. If the final computed hash is different from the pre-selected $h_{0}$, he/she generates a random challenge string $c^{\prime }$. Otherwise, he/she uses the attacker’s PK to encrypt message $m_{b}$ and sends $c\leftarrow Enc(m_{b},PK)$. The attacker’s goal is to recover random bit *b* using the received *c*. He/she wins the game if he/she outputs the correct *b*.

We can see that if the attacker did not provide PK, which the challenger considers authentic, he/she has no information about *b* and can only guess it with a probability of 1/2. Otherwise, he/she might be able to derive some information from *c* and has some advantage in the game, where Adv(A)=Pr(A(c)=b)−1/2. Our scheme is secure if the advantage of any PPT attacker is negligible.

We prove the security of the scheme by contradiction. Let us suppose that there exists an attacker A that has a non-negligible advantage in the security game as defined above. To consistently win the game, the attacker has to be able to:

1. provide PK, which is accepted by the attacker with non-negligible probability (otherwise, his/her advantage would remain negligible due to random challenge strings $c^{\prime }$);
2. distinguish messages encrypted by the provided PK with non-negligible advantage.

If the attacker can distinguish messages, this means that he/she can either break the underlying public key scheme, which breaks the security assumptions we used, or he/she knows the corresponding secret key. Thus, we can conclude that a successful attacker must be able to authenticate key pairs (SK,PK), generated by him/her, to succeed in the security game.

To successfully authenticate the attacker’s PK, the PK with the generated authenticated path ${\left\{h_{i_{j}}\right\}}_{j=1}^{M}$ must produce the pre-determined value $h_{0}$ as the output of hash function hash, with non-negligible probability. However, by reduction, such an attacker can be used to compute preimages of the hash function, and thus violates the security assumptions.

There is one exception to the above argument: if the attacker can reuse (part) of the authentication path, which already exists in the Merkle tree. If the attacker reuses only a part of the path, we still get a reduction to the hash function preimage; in this case, the attacker can use this to compute preimages of some of the hashes stored in the nodes of the Merkle tree. If the attacker uses the whole path from the Merkle tree, this means that he/she can generate such a keypair (SK,PK) that hashes to one of the final leaves in the Merkle tree. If PK is not one of those used to build the Merkle tree, this again violates the preimage resistance of the hash function. Notice that the attacker can succeed (by chance) in constructing a preimage of any of the hashes stored in the Merkle tree. Thus, we need to ensure that the preimage resistance of the used hash function is higher than $2^{\lambda +M}$ to keep the attacker’s advantage below $2^{-\lambda }$.

Finally, the attacker might be able to randomly generate one of the $2^{M}$ precomputed ephemeral public keys used to build the Merkle tree. Due to the security assumption on KDF, this can only happen with probability $2^{M-n}$ (otherwise, the attacker would be able to break KDF, which violates the security assumptions). Again, to keep the attacker’s advantage below $2^{-\lambda }$, we require that n≥M+λ, i.e., we need to use random seeds (pre-keys) for generated keypairs with at least λ+M bits.

To conclude the proof, our scheme provides IND-CPA security with key authentication with security level λ, under the assumption that the underlying public key scheme is already IND-CPA secure (on the same security level), and we use a secure hash function with at least λ+M output bits and a pre-key with a seed of at least λ+M random bits.

## 6. Prototype Implementation of the Protocol

We created an experimental implementation of the proposed protocol in the Master’s thesis [27]. The main aim of the implementation was to verify the feasibility of the protocol on standard computing platforms (simple PC) and estimate the practical height of the Merkle tree suitable for basic Internet servers. The proposed protocol was integrated with TLS 1.2 flow; thus, it is readily available to use in real-world client-server communication.

**Figure 4:**
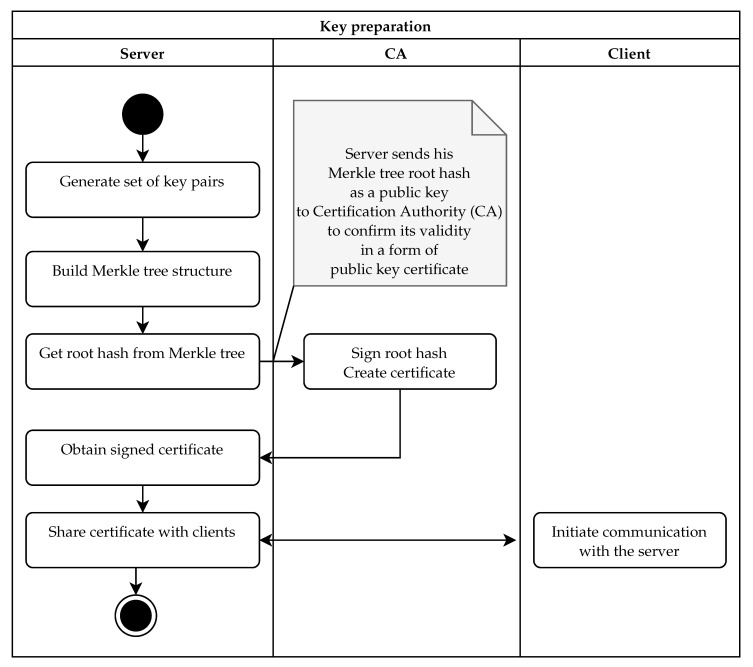
Preparation of keys on the server side [27].

**Figure 5:**
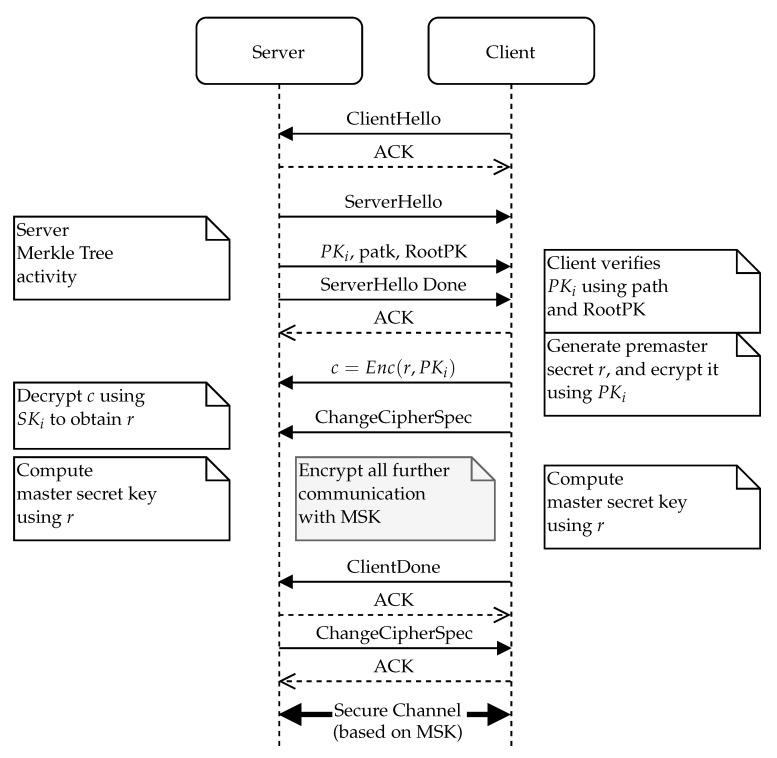
Overview of the protocol use within TLS 1.2 scope for client-server communication [27]. PK, public key.

**Figure 6:**
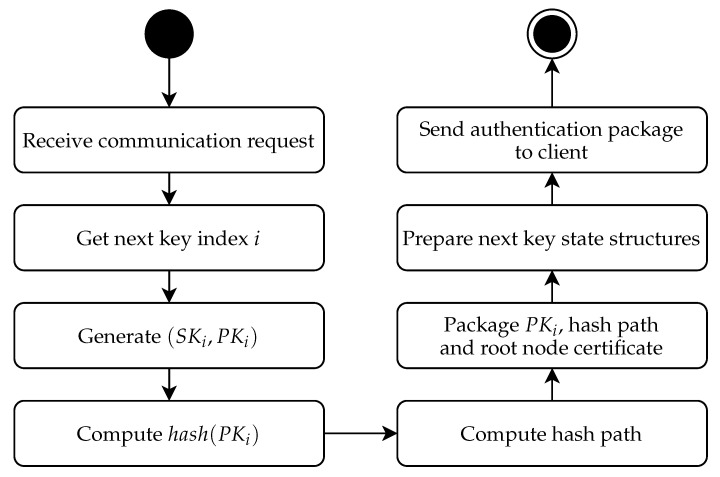
Update of the authentication path for the next ephemeral key on the server [27].

In our implementation of the protocol, the server pre-generates a set of ephemeral keys and the Merkle tree structure that authenticates them (see Figure 4). This part can be done anytime during server initialization, either directly on the server or in dedicated hardware. During the TLS handshake, the server provides an ephemeral key to the client, and the client validates the key using the Merkle tree root (see Figure 5). We use the basic version of the protocol with sequential tree authenticated ephemeral keys. After each key, the server needs to recompute the authentication path for the next key in the series (see Figure 6).

The server preparation phase is detailed in Figure 4. In this case, we use a security model similar to PKI. Thus, we need a dedicated certification authority (CA): a trusted entity in the PKI infrastructure. The workflow of the key preparation is started on the server by generating a set of $2^{l}$ ephemeral key pairs. In the next step, public keys are hashed, and a Merkle tree is built in *l* levels on top of the public key hashes. As a result of this stage, we obtain the Merkle tree and the hash value associated with the root of the Merkle tree. This root hash authenticates the whole set of key pairs, and thus, it plays the role of the long-term authentication public key. In the third step, the root hash is sent to the CA for signing. The CA creates a public key certificate. We do not prescribe any specific algorithm, but if we need long-term post-quantum security, the CA needs to use some specific quantum-resistant signing algorithm. In the final stage, the signed certificate must be installed on the server. This certificate is then shared with each client when the client initiates the TLS communication.

In IoT scenarios with limited resources under the manufacturer’s control, it is possible to skip the PKI and certificates. Instead, the hash stored in the root node of the authentication tree is preinstalled on the manufactured devices. It is then used as a (pinned) server certificate, e.g., when downloading updates. The main advantage is that the server certificate has the smallest possible length (a single hash value) necessary to provide the desired security level.

The main flow of the TLS protocol enhanced with an ephemeral key authenticated with the Merkle tree is presented in Figure 5. As usual, the client starts the protocol with the ClientHello message. After the server receives a ClientHello, it must respond with its public key included in the ServerHello message. During this phase, the server needs to access the key storage and select an ephemeral key that will be used in this particular session. The server computes the ephemeral key’s authentication path. To protect from DoS attacks, the actual authentication path for the next key should be stored and recomputed only once the key is actually used for communication (after finishing the TLS handshake).

The client is presented with the selected ephemeral public key along with its authentication path and the certificate of the Merkle tree root node. The client then must verify the obtained hash path in its entirety and confirm the validity of the Merle tree root’s certificate (if the Merkle tree root is not already pinned on the client device). Once the client has verified the server’s ephemeral public key, it can use this key to send an encrypted key material (e.g., by using the key encapsulation method for a randomly chosen session key).

Finally, the server uses the private key part belonging to the presented ephemeral public key to finalize the underlying symmetric key exchange. Once both parties have the same symmetric key established, they can then communicate with a standard TLS record protocol.

An important part of the enhanced protocol is the state update phase on the server depicted in Figure 6. As discussed earlier, these steps can be done upon a new key request (as depicted in Figure 6) or can be postponed after the communication is established. In the latter case, the trigger is not “receive communication request”, but the trigger event is “previous communication established”.

The “get next key index” procedure depends on whether we are using a sequential (see Section 4.1) or a parallel (see Section 4.2) key structure at the bottom of the Merkle tree. In the case of the sequential structure, the key index is increased by one, and previous keys are purged from the storage if the keys are precomputed (they can be kept in the cache if still needed in active communication). In the case of the parallel structure, the next key index is selected randomly.

Once we have established the next key index, the process of preparing the next key is started, and we must access or generate the actual ephemeral key pair. If the key pairs are precomputed and stored in a secure memory, we just access the key from secure storage to runtime cache. If the actual ephemeral keys are not stored, we generate the next key depending on the key structure. In the case of the sequential key structure, we update the pre-key via a one way function (we use SHA-2 in our experimental software), obtaining the seed for a new ephemeral key. In the case of a parallel key structure, we access the pre-key in the secure storage and generate an ephemeral key seed by hashing it with the new key index.

After having obtained the ephemeral key seed, we use the key generation algorithm from our (post-quantum) public key encryption (or KEM) algorithm to obtain the actual key pair. We then compute the hash of the public key part and the verification path. This can be either done by just accessing the hash nodes in the stored Merkle tree or be recomputed from a previously known hash path. The first option is memory intensive, but very fast; the second option is slow, but can be used in cases where memory is limited. Note that the Merkle tree structure we are storing is not actually secret. Thus, any standard storage can be used for a precomputed Merkle tree (but not the secret keys that underlie the tree).

After a new verification path is computed, it can be packaged and stored on the server cache for handling the next request or directly sent to the client in the case that we are handling the update directly on the communication request. In the case of a server with heavy traffic, we might consider preparing a set number of ephemeral keys along with their authentication paths in advance. This can be done in a background thread. Note that in this case, we need to ensure that private keys are securely stored in the server cache. Note that to prevent exhausting the ephemeral keys quickly, we should also implement the management of TLS sessions, so we do not need to send multiple ephemeral keys to the same client too often (e.g., by reusing pre-shared symmetric keys on reopening the session).

## 7. Experimental Results

To verify our theoretical results, we initiated and supervised thesis [27], where the tree authentication of ephemeral public keys was implemented and tested. Due to the thesis being written in Slovak, in this section, we summarize important experimental results of that work for the sake of completeness of this article.

The implementation was only focused on a sequential version, which is more complicated due to more complex state management. We chose to combine tree authentication with post-quantum KEM BIKE [11]. We used the (open-source) reference implementation from the NIST challenge [28], with a slight change required to generate key pairs from a fixed seed. For the hashing and KDF, we used the SHA-256 implementation from the OpenSSL library, Version 1.1.0g.

The following tests were run on a PC with processor Intel(R) Core(TM) i7-4712MQ CPU @ 2.30 GHz. The operating memory size was 8 GB. The testing PC can compute 75 million SHA-256 hashes per second (using 16 B input blocks, tested using the openssl speed command).

We ran the experiments with three sizes of the Merkle tree: l=18, l=21, and l=24, respectively. Every time measurement is an average from 10 (randomized) experiments. Table 1 shows the total time required for individual operations. Keypair generation is the time required to compute $2^{l-1}$ individual BIKE key pairs (only a basic BIKE-1 key generation algorithm was used; no batch generation). After that, the Merkle tree building consisted of hashing public keys and computing hashes in the tree nodes. In our experiments, the whole Merkle tree was always stored in the RAM of the experimental computer. Finally, we ran through the signature generation for all public keys and one verification of the signature per key. Note that signature generation is faster than signature verification because we stored the whole Merkle tree in the memory. If we re-computed (part of the) Merkle tree for each signature, the total time for a single signature would be upper bounded by the Merkle tree building time (for all keys).

To normalize the times between different sizes of the Merkle tree, we computed the average time per key in Table 2. We can see that the cost of building the Merkle tree is amortized when storing the tree in memory, and both signature generation and signature verification are small compared to the key generation algorithm. Note that if we re-generated the whole tree for each signature, the (maximum) time for creating the key signature would grow quickly. We do recommend storing the whole Merkle tree in the memory once it is generated.

The maximum level that was possible to store in the 8GB of memory on our testing PC was l=26 (approximately 30 million key pairs). The BIKE key generation for this level took 2.5 h of real-time computation (note that the sequential key generation cannot be sped up by parallel computing). Once the keys were generated, the building of a Merkle tree was fast: it took only 17 s of computational time. Thus, in a scenario with a busy server requiring a large number of ephemeral keys, these are preferably generated in advance, and parallel storage (see Figure 3) is preferred (note that the server might lose the forward security property; see Section 4.2).

## 8. Discussion

Public key encryption schemes (or KEMs) with ephemeral keys only (or keys with a limited number of uses) are sufficient to build TLS-like handshake protocols. While the authentication of the protocol can be solved with an additional signature scheme, we can also authenticate the ephemeral public keys directly by employing the Merkle tree technique. In a post-quantum setting, this technique can provide a building block to more efficient and flexible authenticated key establishment schemes.

Selected components and their well-known security properties should provide tools to create a key exchange mechanism (with underlying KEM) with server authentication (through the use of Merkle tree-based signatures) and, optionally, forward secrecy (by the use of ephemeral keys). If our use case requires a client authentication as well, we can also precompute ephemeral keys and the related Merkle tree on the client device. The client then registers his/her device with the Merkle tree root (which has the role of the client’s public key) and presents the server with an ephemeral public key and authentication path in the key exchange phase of the TLS protocol.

The main advantage of the client keys is that both sides of the communication are authenticated, and we do not need to implement other authentication measures at the application level. It is not necessary to prevent man-in-the-middle attacks (server certificate is sufficient). Note that the client certificate does not protect the client device against a key compromise. However, if client-side authentication is employed, the server has more control over the connections. While the compromised client device can, e.g., try to exhaust keys by launching many connection requests, the server can employ higher level controls, such as monitoring the number of connection attempts, to detect and block the malicious client behavior.

In the TLS setting, the lifetime of the set of authenticated keys can also be extended by using pre-shared keys for clients that re-connect to the server and with suitable use of hierarchical PKI.

Our experimental results confirm the feasibility of the scheme in practice. For the selected KEM (algorithm BIKE), the most difficult part was the key generation, which is also mandatory in any scheme with ephemeral keys regardless of the chosen authentication method. Merkle tree building took only a very small fraction (less than 0.2%) of the key generation time (per key).

Signature generation, however, is fast only if the whole Merkle tree is precomputed in the memory. The Merkle tree is not secret; thus, there is no need for a special protected memory to store it (unlike the master secret key or the precomputed secret keys). In our experimental PC with 8 GB of memory, we were able to store a tree of level l=26 without negatively affecting the performance of the computer. This means that such a Merkle tree can authenticate approximately 30 million key pairs, which allows almost 46 thousand active connections per day on average for two years. On the client-side, we can use a smaller Merkle tree (with a lower number of levels *l*), depending on the expected number of connections the client will be doing in the lifetime of the authentication key, and the resources available.

Due to very fast signature generation and easy verification on the client (short public key, short signature, fast verification using just the standard hash function), this scheme can be very useful for IoT applications. If the number of clients or connections is limited or known in advance, we can optimize the parameters (mostly the height of the tree *l*) used on the server: if we increase *l*, we increase the number of connections available during the lifetime, but also (slightly) increase the length of the signature and the complexity of the key verification. Note that the Merkle tree has an advantage over directly storing a limited set of the server’s ephemeral public key hashes on the client. We save the storage required on the client (a single Merkle tree root takes less space than multiple public key hashes). Furthermore, if the number of connections of various clients is not uniform, we are not limited by the expected maximum number of connections any client might require, but only by an expected total number of connections of all clients during the lifetime of the server’s public key.

On the other hand, the fixed number of authenticated keys pre-authenticated with the Merkle tree limits the use of the scheme in servers with heavy traffic. In such a case, we recommend using the parallel version of the protocol with possible key reuse (should be limited based on the security restrictions of the underlying KEM and requirements of possible forward security).

## 9. Summary

We propose a novel protocol that employs Merkle trees to provide post-quantum authentication for a set of ephemeral keys. Our scheme has two variants. The sequential variant uses a single pre-key from which ephemeral keys are derived in a one way sequence. This variant provides inherent forward secrecy: keys used in the past cannot be recovered. On the other hand, session management is more complicated, as the ephemeral keys can only be accessed in a sequence. The parallel variant uses a master key that can derive any pre-authenticated ephemeral key. This allows us to enable parallel sessions and possible key re-use (if allowed by underlying key exchange mechanism), but removes forward secrecy. Forward secrecy for the parallel version can be implemented at the cost of secure storage by precomputing and storing all ephemeral keys.

Furthermore, we experimentally verified the feasibility of the proposed protocol, by instantiating it with the BIKE KEM algorithm and the corresponding key generator, as well as the SHA2 function as a one way function. Our experiments show that the cost of the Merkle tree setup and use takes less than 0.2% of the time compared to overall key generation. It is also favorable in comparison to using one of the proposed post-quantum signature algorithms, mainly because we do not need an extra signature key (we replace the public key with the Merkle tree root and the private key with the knowledge of the ephemeral private key, or the pre-key, respectively). The running time and memory requirements show that Merkle tree authentication is suitable for deployment on a server and PC-like clients, but its use might be limited in restricted devices. On the other hand, IoT clients can verify the server they are connecting to very quickly using only a standard hash function.

## Figures and Tables

**Table 1:** Total time required to run an individual algorithm in the protocol.

	Total Time for All Keys (ms)		
Merkle Tree Levels	18	21	24
No. of Keypairs	$2^{17}$	$2^{20}$	$2^{23}$
Keypair Generation	35,442	285,894	2,254,612
Merkle Tree Building	57	451	3662
Signature Generation	188	1821	16,819
Signature Verification	971	9085	82,695

**Table 2:** Average time required to run an individual algorithm in the protocol (per keypair).

	Total Time for All Keys (μs)		
Merkle Tree Levels	18	21	24
No. of Keypairs	$2^{17}$	$2^{20}$	$2^{23}$
Keypair Generation	270.402	272.650	268.771
Merkle Tree Building	0.437	0.431	0.437
Signature Generation	1.440	1.737	2.005
Signature Verification	7.412	8.665	9.858

## Data Availability

The data presented were generated by experimental software available as a supplementary material of [27].
